# Supplementary material for: Post-COVID spirometric abnormalities in workers with intermittent high-altitude exposure: A cross-sectional study in Peru
Source: PLoS One. 2026 May 18;21(5):e0329054. doi: 10.1371/journal.pone.0329054 (PMC13183208; doi:10.1371/journal.pone.0329054)
Supplement: S1 Table — (DOCX) [file pone.0329054.s001.docx]

### **S1 Table. Frequency of Spirometric Patterns by Clinical and Occupational Characteristics**

| **Variable** | **Normal Spirometry**  **n (%)** | **Obstructive Pattern**  **n (%)** | **Restrictive Pattern**  **n (%)** | **Restrictive/**  **Obstructive Pattern**  **n (%)** |
| --- | --- | --- | --- | --- |
| **Sex** |  |  |  |  |
| Male | 53 (47.8) | 29 (65.9) | 45 (54.2) | 85 (52.5) |
| Female | 58 (52.2) | 15 (34.1) | 38 (45.8) | 77 (47.5) |
| **Age** |  |  |  |  |
| < 40 years | 33 (29.7) | 16 (36.4) | 31 (37.4) | 55 (33.9) |
| 40 – 49 years | 29 (26.1) | 11 (25.0) | 17 (20.5) | 43 (26.5) |
| 50 – 59 years | 39 (35.1) | 9 (20.5) | 24 (28.9) | 43 (26.5) |
| 60 – 65 years | 10 (9.0) | 8 (18.2) | 11 (13.2) | 21 (12.9) |
| **Body Mass Index (BMI)** |  |  |  |  |
| Normal (<25 kg/m²) | 42 (37.9) | 14 (31.8) | 21 (25.3) | 57 (35.2) |
| Overweight (25–29.9 kg/m²) | 69 (62.1) | 15 (34.1) | 32 (38.6) | 68 (42.0) |
| Obesity (≥30 kg/m²) | 0 (0) | 15 (14.1) | 30 (36.1) | 37 (22.8) |
| **Charlson Comorbidity Index** |  |  |  |  |
| No comorbidities | 70 (63.1) | 20 (45.5) | 26 (31.3) | 73 (45.1) |
| Low burden (Charlson Index 1 or 2) | 41 (36.9) | 7 (15.9) | 26 (31.3) | 51 (31.4) |
| High burden (Charlson Index ≥3) | 0 (0) | 17 (38.6) | 31 (37.4) | 38 (23.5) |
| **COVID-19 Severity** |  |  |  |  |
| Outpatient management | 69 (62.2) | 27 (61.4) | 50 (60.2) | 95 (58.6) |
| General hospitalization | 42 (37.8) | 14 (31.8) | 24 (28.9) | 44 (27.2) |
| Intensive care | 0 (0) | 3 (6.8) | 9 (10.8) | 23 (14.2) |
| **Occupation** |  |  |  |  |
| Administrative | 21 (18.9) | 4 (9.1) | 14 (16.9) | 30 (18.5) |
| Supervisor | 20 (18.0) | 7 (15.9) | 13 (15.7) | 24 (14.8) |
| Environmental health and safety | 37 (33.3) | 18 (40.9) | 31 (37.4) | 42 (32.1) |
| Technician | 16 (14.4) | 5 (11.4) | 14 (16.9) | 28 (17.3) |
| Operator | 17 (15.3) | 10 (22.7) | 11 (13.3) | 28 (17.3) |
| **Duration of Employment** |  |  |  |  |
| 3 to 4.9 years | 38 (34.2) | 24 (54.6) | 34 (41.0) | 48 (29.6) |
| 5 to 6.9 years | 33 (29.7) | 8 (18.2) | 25 (30.1) | 57 (35.2) |
| ≥ 7 years | 40 (36.0) | 12 (27.3) | 24 (28.9) | 57 (35.2) |
| **Prior Occupational Exposure to Respiratory Irritants** |  |  |  |  |
| None | 65 (58.6) | 17 (38.6) | 31 (37.4) | 73 (45.1) |
| 1 to 3 years | 22 (19.8) | 10 (22.7) | 18 (21.6) | 37 (22.8) |
| 3 to 5 years | 24 (21.6) | 12 (27.3) | 20 (24.1) | 32 (19.8) |
| ≥ 5 years | 0 (0) | 5 (11.4) | 14 (16.9) | 20 (12.4) |
| **Intermittent High-Altitude Exposure** |  |  |  |  |
| < 3 years | 71 (64.0) | 15 (34.1) | 29 (34.9) | 50 (30.9) |
| 4 to 6 years | 40 (36.0) | 15 (34.1) | 25 (30.2) | 40 (24.6) |
| ≥ 7 years | 0 (0) | 14 (31.8) | 29 (34.9) | 72 (44.5) |

Percentages represent the proportion of each spirometric pattern within every clinical or occupational category. Ventilatory patterns were classified according to ATS/ERS 2021 standards (obstructive: FEV₁/FVC < 0.70; restrictive: FVC < 80% predicted with preserved ratio; mixed: both reduced). Only A/B–quality spirometry was included.
