# Supplementary material for: Post-COVID spirometric abnormalities in workers with intermittent high-altitude exposure: A cross-sectional study in Peru
Source: PLoS One. 2026 May 18;21(5):e0329054. doi: 10.1371/journal.pone.0329054 (PMC13183208; doi:10.1371/journal.pone.0329054)
Supplement: S2 Table — (DOCX) [file pone.0329054.s002.docx]

**S2 Table. Spirometric Indices (FEV₁, FVC, and FEV₁/FVC Ratio) by Demographic, Clinical, and Occupational Characteristics"**

| **Category** | **VEF1**  **Mean ± SD** | **VEF1**  P50 [p25 to p75 | **CVF**  **Mean ± SD** | **CVF**  P50 [p25 to p75] | **FEV₁/FVC Ratio**  **Mean ± SD** | **FEV₁/FVC Ratio**  P50 [p25 to p75 |
| --- | --- | --- | --- | --- | --- | --- |
| **General** | 65.20 ± 18.71 | 61.49 [47.46 to 82.27] | 0.803 ± 0.077 | 0.784 [0.745 to 0.862] | 80.98 ± 21.77 | 69.64 [62.04 to 98.88] |
| **Age** |  |  |  |  |  |  |
| < 40 years | 65.07 ± 18.99 | 60.33 [47.58 to 82.98] | 0.801 ± 0.070 | 0.786 [0.750 to 0.847] | 81.04 ± 22.52 | 69.17 [62.45 to 99.88] |
| 40 – 49 years | 65.35 ± 19.60 | 58.70 [47.05 to 85.47] | 0.810 ± 0.086 | 0.778 [0.751 to 0.874] | 80.40 ± 22.52 | 69.11 [61.28 to 97.80] |
| 50 – 59 years | 66.10 ± 17.92 | 73.27 [48.09 to 81.55] | 0.801 ± 0.078 | 0.779 [0.735 to 0.863] | 82.19 ± 20.24 | 86.12 [63.03 to 99.78] |
| 60 – 65 years | 63.21 ± 18.28 | 57.02 [46.60 to 79.55] | 0.798 ± 0.071 | 0.784 [0.747 to 0.856] | 79.17 ± 22.06 | 67.13 [61.79 to 93.80] |
| **Sex** |  |  |  |  |  |  |
| Female | 66.56 ± 19.28 | 69.40 [47.46 to 84.53] | 0.807 ± 0.079 | 0.784 [0.746 to 0.868] | 82.16 ± 21.96 | 74.03 [62.24 to 99.52] |
| Male | 64.00 ± 18.15 | 58.39 [47.41 to 81.03] | 0.799 ± 0.074 | 0.783 [0.741 to 0.854] | 79.92 ± 21.59 | 68.67 [61.72 to 98.66] |
| **Body Mass Index (BMI)** |  |  |  |  |  |  |
| Normal (< 25 kg/m²) | 64.62 ± 19.66 | 59.53 [46.24 to 83.53] | 0.805 ± 0.079 | 0.783 [0.745 to 0.864] | 79.72 ± 21.79 | 69.16 [60.81 to 99.27] |
| Overweight (25.0–29.9 kg/m²) | 67.75 ± 18.57 | 75.84 [48.23 to 82.95] | 0.813 ± 0.080 | 0.793 [0.746 to 0.874] | 83.10 ± 21.18 | 86.25 [63.34 to 98.88] |
| Obesity (≥ 30 kg/m²) | 60.44 ± 16.48 | 54.22 [47.29 to 75.56] | 0.777 ± 0.055 | 0.767 [0.737 to 0.792] | 78.25 ± 22.83 | 66.56 [61.89 to 98.33] |
| **Charlson Comorbidity Index** |  |  |  |  |  |  |
| No comorbidities | 66.15 ± 18.98 | 69.80 [47.83 to 83.00] | 0.815 ± 0.080 | 0.795 [0.754 to 0.875] | 80.70 ± 20.88 | 76.15 [62.45 to 97.98] |
| Low burden (Charlson Index 1 or 2) | 66.85 ± 19.32 | 71.53 [47.69 to 84.11] | 0.803 ± 0.079 | 0.778 [0.744 to 0.856] | 82.91 ± 22.00 | 82.38 [62.76 to 100.44] |
| High burden (Charlson Index ≥3) | 60.73 ± 16.59 | 54.16 [47.00 to 78.58] | 0.777 ± 0.058 | 0.761 [0.738 to 0.787] | 78.77 ± 23.30 | 66.36 [60.62 to 100.86] |
| **COVID-19 Severity** |  |  |  |  |  |  |
| Outpatient management | 65.65 ± 18.92 | 63.35 [47.34 to 83.05] | 0.803 ± 0.079 | 0.782 [0.742 to 0.860] | 81.53 ± 21.97 | 69.88 [62.01 to 99.72] |
| General hospitalization | 66.95 ± 18.12 | 72.30 [49.20 to 81.80] | 0.814 ± 0.075 | 0.792 [0.754 to 0.873] | 81.98 ± 20.65 | 82.02 [63.89 to 98.57] |
| Intensive care | 55.95 ± 17.06 | 47.96 [45.17 to 66.50] | 0.763 ± 0.044 | 0.757 [0.734 to 0.785] | 73.66 ± 23.47 | 62.64 [59.42 to 91.86] |
| **Occupation** |  |  |  |  |  |  |
| Administrative | 65.73 ± 20.23 | 69.01 [47.34 to 86.02] | 0.802 ± 0.073 | 0.782 [0.750 to 0.854] | 81.46 ± 22.91 | 82.38 [61.80 to 99.62] |
| Supervisor | 65.34 ± 19.00 | 62.34 [47.71 to 82.43] | 0.805 ± 0.078 | 0.786 [0.743 to 0.867] | 81.05 ± 22.68 | 69.67 [61.52 to 100.44] |
| Environmental Health and Safety | 63.48 ± 18.10 | 59.26 [46.60 to 80.98] | 0.807 ± 0.078 | 0.783 [0.747 to 0.866] | 78.22 ± 19.92 | 68.62 [62.07 to 93.94] |
| Technician | 66.25 ± 17.64 | 66.95 [48.28 to 81.67] | 0.799 ± 0.069 | 0.781 [0.745 to 0.848] | 82.74 ± 20.45 | 80.69 [63.11 to 98.90] |
| Operator | 65.08 ± 18.47 | 59.61 [46.51 to 81.33] | 0.800 ± 0.085 | 0.773 [0.736 to 0.852] | 81.36 ± 22.02 | 69.17 [60.89 to 99.27] |
| **Duration of Employment in Current Company** |  |  |  |  |  |  |
| 3 to 4.9 years | 66.15 ± 17.50 | 64.84 [49.94 to 81.46] | 0.810 ± 0.076 | 0.787 [0.749 to 0.869] | 81.79 ± 21.30 | 70.91 [62.70 to 98.84] |
| 5 to 6.9 years | 64.70 ± 19.39 | 57.26 [46.56 to 83.90] | 0.794 ± 0.079 | 0.773 [0.734 to 0.842] | 81.03 ± 22.00 | 69.15 [62.45 to 99.88] |
| ≥ 7 years | 64.65 ± 19.42 | 61.23 [46.42 to 82.18] | 0.804 ± 0.075 | 0.784 [0.749 to 0.860] | 80.05 ± 22.18 | 69.20 [60.77 to 98.33] |
| **Prior Occupational Exposure to Respiratory Irritants** |  |  |  |  |  |  |
| No | 66.54 ± 18.75 | 70.58 [47.91 to 82.49] | 0.810 ± 0.081 | 0.784 [0.746 to 0.868] | 81.72 ± 20.75 | 79.84 [62.64 to 98.52] |
| 1 to 2.9 years | 64.30 ± 18.87 | 57.91 [46.51 to 83.00] | 0.799 ± 0.074 | 0.787 [0.745 to 0.853] | 80.10 ± 21.58 | 69.17 [62.77 to 99.27] |
| 3 to 4.9 years | 65.58 ± 18.97 | 64.36 [47.33 to 82.21] | 0.808 ± 0.078 | 0.789 [0.749 to 0.869] | 81.25 ± 23.19 | 70.81 [60.50 to 100.32] |
| ≥ 5 years | 59.98 ± 17.17 | 53.14 [46.52 to 77.41] | 0.766 ± 0.047 | 0.759 [0.730 to 0.785] | 78.76 ± 24.14 | 66.95 [61.45 to 100.44] |
| **Intermittent High-Altitude Exposure** |  |  |  |  |  |  |
| < 4 years | 69.49 ± 18.21 | 76.67 [49.94 to 84.97] | 0.824 ± 0.085 | 0.808 [0.751 to 0.876] | 84.04 ± 20.04 | 89.46 [64.55 to 99.27] |
| 4 to 6.9 years | 67.47 ± 18.20 | 74.22 [48.53 to 81.86] | 0.809 ± 0.072 | 0.796 [0.752 to 0.859] | 83.19 ± 21.09 | 88.88 [62.33 to 100.25] |
| ≥ 7 years | 56.68 ± 17.23 | 49.83 [45.63 to 66.17] | 0.766 ± 0.051 | 0.758 [0.728 to 0.783] | 74.27 ± 23.50 | 65.12 [59.83 to 89.04] |

Values are presented as mean ± standard deviation and as median with interquartile range (P50 [P25–P75]). Spirometric indices (FEV₁, FVC, and FEV₁/FVC ratio) were interpreted according to ATS/ERS 2019–2021 standards, and only A/B–quality tests were included. FEV₁ = Forced Expiratory Volume in the first second; FVC = Forced Vital Capacity
