# Supplementary material for: Post-COVID spirometric abnormalities in workers with intermittent high-altitude exposure: A cross-sectional study in Peru
Source: PLoS One. 2026 May 18;21(5):e0329054. doi: 10.1371/journal.pone.0329054 (PMC13183208; doi:10.1371/journal.pone.0329054)
Supplement: Attachment — Submitted filename: Response to reviewers - R1.docx [file pone.0329054.s005.docx]

**Response to Reviewers**

Manuscript ID:
Title: Post-COVID Spirometric Abnormalities in Workers with Intermittent High-Altitude Exposure: A Cross-Sectional Study in Peru

Dear Editor and Reviewers,

We sincerely thank you for the thorough and constructive feedback provided on our manuscript. Each comment has been carefully considered and addressed to improve clarity, scientific rigor, and full compliance with PLOS ONE editorial requirements. The revised version includes a substantially strengthened Discussion section, an expanded and fully detailed Ethics Statement, a more informative Abstract, and smoother transitions throughout the manuscript to enhance narrative coherence.

Below, we present a point-by-point response to all editorial and reviewer observations, along with a concise description of the corresponding revisions implemented in the manuscript. All modifications have been incorporated into both the tracked-changes version and the clean version submitted.

We are grateful to the reviewers and the editorial team for their insightful comments, which have significantly enhanced the quality, clarity, and robustness of our work.

Sincerely,
Marlon Yovera-Aldana

EDITORIAL REQUIREMENTS

1. **Please ensure that your manuscript meets PLOS ONE's style requirements, including those for file naming.**

Response: We appreciate this reminder. The manuscript has been revised to fully comply with PLOS ONE formatting requirements. Specifically: (1) the main text now follows the structure and stylistic conventions indicated in the PLOS ONE sample templates; (2) the title page, author list, and affiliations have been reformatted according to journal specifications; and (3) all files—main manuscript, figures, and supplementary materials—have been renamed following PLOS ONE’s file-naming guidelines. We have reviewed all submission components to ensure consistency with the journal’s style standards.

2. **Please provide additional details regarding participant consent. In the ethics statement in the Methods and online submission information, please ensure that you have specified (1) whether consent was informed and (2) what type you obtained (for instance, written or verbal, and if verbal, how it was documented and witnessed). If your study included minors, state whether you obtained consent from parents or guardians. If the need for consent was waived by the ethics committee, please include this information.**

**If you are reporting a retrospective study of medical records or archived samples, please ensure that you have discussed whether all data were fully anonymized before you accessed them and/or whether the IRB or ethics committee waived the requirement for informed consent. If patients provided informed written consent to have data from their medical records used in research, please include this information.

We appreciate the request for clarification. We confirm that the manuscript already incorporates all required ethical details regarding consent and data handling. Specifically, the Ethics Statement explains that:**

The study used secondary, anonymized data obtained from routine occupational health evaluations.

No identifiable personal information was accessible to the research team.

The Institutional Committee of Ethics in Research (CIEI) of Universidad Científica del Sur reviewed and approved the study (CONSTANCIA N°670-CIEI-CIENTÍFICA-2024; protocol POS-60-2024-00841).

The CIEI waived the requirement for individual informed consent, in accordance with national regulations, because all data were fully anonymized prior to analysis and the study posed minimal risk.

No minors were included.

3. **Please include captions for your Supporting Information files at the end of your manuscript, and update any in-text citations to match accordingly. Please see our Supporting Information guidelines for more information:** [**http://journals.plos.org/plosone/s/supporting-information**](http://journals.plos.org/plosone/s/supporting-information)**.**

We appreciate this clarification. We have now added full captions for all Supporting Information files (S1 Table and S2 Table) at the end of the manuscript, following PLOS ONE formatting guidelines. In addition, all in-text citations referring to these files have been updated to ensure consistency with the final Supporting Information labeling

Reviewer #1:

**1. Novelty and Contribution**

**The study’s main strength is the inclusion of pre-pandemic spirometry, allowing a before–after perspective rarely available in post-COVID occupational studies.**

**However, the novelty is somewhat diluted in the Introduction and Discussion. The manuscript should emphasize more clearly:**

**A. That most prior studies lacked pre-COVID baselines.**

**B. That very few have studied intermittent high-altitude exposure.**

**Recommendation: Strengthen these points in the Introduction (last paragraph) and Discussion (main findings + conclusion).**

We appreciate the reviewer’s observation. We have strengthened the manuscript to more clearly highlight the two main novel aspects of the study:

(1) the availability of pre-pandemic spirometry, which is uncommon in post-COVID research and enables a true before–after comparison, and

(2) the limited evidence on post-COVID pulmonary function among workers with intermittent high-altitude exposure.

These points have been explicitly emphasized in the Introduction (final paragraph), the Discussion (Main Findings), and the Conclusion.

2. **Study Design and Methods**

**The cross-sectional design is acknowledged, but since pre-COVID spirometry exists, the study has elements of a retrospective cohort. Clarify this framing: Was the analysis truly cross-sectional, or longitudinal with before–after comparison?**

**Spirometry interpretation: It is not fully clear whether ATS/ERS standards were followed for quality control and reproducibility. Please clarify in Methods.**

**Occupational exposure assessment: More detail is needed on how “inhalational exposure” was defined (e.g., type of dusts/fumes, self-reported vs. workplace records). Right now it reads too broadly.**

We appreciate the reviewer’s request for clarification. Although pre-pandemic spirometry was available, the analysis was conducted as a cross-sectional study because no longitudinal change or within-subject comparisons were modeled. The pre-COVID spirometry was used exclusively to confirm normal lung function at baseline as part of the eligibility criteria. This clarification has been added to the Study Design subsection.

We have also expanded the Methods to explicitly state that spirometry procedures followed ATS/ERS 2019 standards for acceptability, reproducibility, and quality grading.

Finally, we have detailed how inhalational exposure was defined, including the specific types of occupational agents considered and the data sources used (occupational health records, job-task documentation, and structured self-report).

3. **Results**

**Well presented, but overly detailed in the text. Many percentages are repeated verbatim from tables. This reduces readability.**

**Suggestion: Condense textual results to highlight only key findings, and let tables carry the detailed breakdown.**

**Example: Instead of listing every FEV₁ group, summarize broadly (“approximately half retained preserved function, while one-third showed moderate impairment”)**

**This would shorten the manuscript and improve flow.**

We thank the reviewer for this recommendation. The Results section has been substantially revised to eliminate redundant numerical descriptions and improve narrative flow. Textual summaries corresponding to Tables 1, 2, 3, and 4 were rewritten to highlight only the key patterns and associations, avoiding repetition of percentages or category-level distributions already displayed in the tables. Detailed values now remain exclusively within the tables.

Changes to the manuscript:

Table 1 (Participant Characteristics): The descriptive narrative was condensed to report only major demographic and clinical trends using qualitative quantifiers (e.g., “two thirds,” “one in five”), without repeating specific percentages.

Table 2 (Spirometric Findings): The text was reduced to emphasize the predominance of abnormal spirometry and the relative distribution of ventilatory patterns, without listing each FEV₁, FVC, or FEV₁/FVC subgroup.

Tables 3 and 4 (Bivariate and Multivariable Analysis): The two sections were merged into a single concise narrative summarizing only the variables that showed meaningful associations, while removing all numerical duplication.

4. **Discussion**

**Comparison with other studies is comprehensive but reads like a series of disjointed summaries. Transitions between sea-level and high-altitude comparisons could be smoothed for narrative coherence.**

**Interpretation section is insightful but somewhat verbose. The mechanistic explanations (airway, interstitial, occupational toxins, hypoxia) could be merged into one integrated paragraph. Similarly, risk factor discussion could be streamlined.**

**At times, speculative mechanisms (e.g., immune dysregulation, environmental synergy) are presented without sufficient qualification. Consider framing these more cautiously.**

**The finding of higher impairment among males compared to females is intriguing but underdeveloped. The explanation (longer altitude exposure, smoking, occupational tasks) is plausible but speculative. Consider reframing more cautiously and noting that sex-specific physiological adaptations at altitude may also contribute.**

We thank the reviewer for these insightful observations. The Discussion section was revised to improve coherence and eliminate fragmented presentation of prior studies. The comparison between sea-level and high-altitude evidence was reorganized to form a continuous narrative, and transitional elements were added to ensure smoother integration of findings across contexts. The Interpretation section was condensed, and the mechanistic explanations related to airway involvement, interstitial processes, occupational inhalational exposures, and hypobaric hypoxia were merged into a single integrated paragraph. The section describing previously reported risk factors was streamlined to remove enumerative structures and present a concise synthesis aligned with the literature. Statements that could be interpreted as speculative were reframed using qualified language, and mechanisms without direct supporting evidence were presented cautiously. The paragraph addressing sex differences was revised to indicate that the crude association did not persist after adjustment, to reference known physiological differences in lung function between males and females, and to note that altitude-related sex-specific adaptations may contribute. Explanations lacking empirical support were removed

.

5. **Public Health & Policy Implications**

**Strong and well-articulated. The suggestion for incorporating spirometry into routine surveillance is practical and impactful.**

**Recommendation: Move this section closer to the Conclusion for emphasis, rather than embedding it between Interpretation and Strengths/Limitations.**

**The occupational recommendations are strong, but the authors could be more explicit about feasibility in low-resource settings (e.g., periodic spirometry may be unrealistic in remote mines). Consider suggesting a stepwise approach (symptom screening, portable spirometry, referral pathways).**

The section on public health and policy implications was relocated closer to the Conclusion, as recommended. The text was revised to explicitly address feasibility challenges in low-resource and remote high-altitude occupational settings. A stepwise approach to surveillance—beginning with symptom screening, followed by portable or simplified spirometry, and referral for full evaluation when indicated—was added. The revised version also clarifies how these measures can be operationalized within existing occupational health systems.

6. **Limitations**

**Appropriate and balanced. However, a limitation worth adding: potential healthy worker effect bias (workers with severe disease or disability may have already exited the workforce, leading to underestimation of true burden). Suggest also noting lack of DLCO or HRCT as a limitation in differentiating restriction vs. diffusion impairment.**

We appreciate the reviewer’s insightful suggestions. The Limitations section was revised to include two additional constraints highlighted by the reviewer. First, the potential for a healthy worker effect was added, acknowledging that workers with severe post-COVID sequelae may have exited the workforce, resulting in possible underestimation of impairment. Second, the absence of DLCO and HRCT, which limits differentiation between restrictive and diffusion abnormalities, was further emphasized..

7. **Conclusion**

**Clear but could be more concise and impactful. Currently too wordy. Aim for a single, powerful paragraph emphasizing:**

**High prevalence (>70%)**

**Independent risk factors**

**Implications for surveillance and policy**

We appreciate the reviewer’s suggestion. The Conclusion section has been revised to be more concise and impactful, focusing on the key elements highlighted: the high prevalence of post-COVID spirometric abnormalities, the main independent risk factors, and the implications for occupational surveillance and policy

Minor Comments

**1. Abstract: Too dense. Consider cutting 30–40 words, focusing on the main result (72.2% prevalence, key predictors).**

The abstract has been streamlined by reducing approximately 35 words, prioritizing the main findings (72.2% prevalence of spirometric abnormalities and key independent predictors).

**2. Terminology: Ensure consistent use of spirometric abnormality vs. pulmonary impairment vs. functional abnormality**.

We have now standardized the wording throughout the manuscript. The primary outcome is consistently referred to as “abnormal spirometry” or “spirometric abnormalities,” in line with the ATS/ERS–based definition provided in the Methods section. Expressions such as “pulmonary impairment” or “functional abnormality” have been either replaced with “abnormal spirometry” when referring to our outcome, or rephrased as “lung function impairment” or “pulmonary sequelae” only when discussing broader clinical consequences beyond spirometric patterns

**3. Numbers consistency: Minor discrepancies (72.2% vs. 72.3%) should be unified.**

We corrected the discrepancy by adopting a single value (72.2%) across the entire manuscript.

4**. References: Overall strong, but some older references could be replaced with more recent 2023–2025 updates if available.**

We thank the reviewer for this helpful suggestion. We have carefully updated the reference list, prioritizing recent (2023–2025) evidence, especially for topics related to COVID-19. In the Introduction, the opening paragraphs now cite recent systematic reviews and cohort studies on long-term post-COVID respiratory sequelae (e.g. Iversen et al., 2025; Ribeiro Carvalho et al., 2024; Salmam et al., 2025), replacing older COVID-19 references. In the Discussion, particularly in the “Comparison with other studies” subsection, we have replaced earlier meta-analyses and cohort data with more recent high-quality studies reporting spirometric and DLCO abnormalities over 6–24 months of follow-up and at different altitudes (e.g. Lee et al., 2022; Chimera et al., 2025; Suri et al., 2024; Gochicoa-Rangel et al., 2023; Chai et al., 2024; Sultana et al., 2025).

Where older references remain (e.g. ATS/ERS technical standards or seminal methodological papers), they were retained because no newer guideline or equivalently comprehensive update is currently available

**5. Statistical Reporting**

**In logistic regression, clarify whether multicollinearity was checked. Some predictors (BMI, comorbidities, duration at altitude) may be correlated.**

**State explicitly whether continuous predictors were analyzed as continuous or categorized.**

We appreciate this observation. Multicollinearity was formally assessed using uncentered Variance Inflation Factors (VIF). All variables showed VIF values between 1.12 and 2.12, with a mean VIF of 1.54, indicating absence of problematic multicollinearity.

All continuous predictors were analyzed as categorical variables, consistent with clinical cut-offs and the distribution of the data. Specifically, age, BMI, Charlson comorbidity index, COVID-19 severity, duration of high-altitude exposure, and inhalant exposure were entered into the model as predefined ordered categories.
